# Optical Addressing Electronic Tongue Based on Low Selective Photovoltaic Transducer with Nanoporous Silicon Layer

**DOI:** 10.1186/s11671-016-1589-0

**Published:** 2016-08-23

**Authors:** S. V. Litvinenko, D. O. Bielobrov, V. Lysenko, V. A. Skryshevsky

**Affiliations:** 1 Institute of High Technologies, Taras Shevchenko National University of Kyiv, 64, Volodymyrs’ka St., 01601 Kyiv, Ukraine; 2 Nanotechnology Institute of Lyon (INL) UMR 5270, CNRS, INSA, University of Lyon, Lyon, Villeurbanne 69621 France

**Keywords:** Electronic tongue, Photovoltaic transducer, Principal component analysis, Porous silicon

## Abstract

The electronic tongue based on the array of low selective photovoltaic (PV) sensors and principal component analysis is proposed for detection of various alcohol solutions. A sensor array is created at the forming of p-n junction on silicon wafer with porous silicon layer on the opposite side. A dynamical set of sensors is formed due to the inhomogeneous distribution of the surface recombination rate at this porous silicon side. The sensitive to molecular adsorption photocurrent is induced at the scanning of this side by laser beam. Water, ethanol, iso-propanol, and their mixtures were selected for testing. It is shown that the use of the random dispersion of surface recombination rates on different spots of the rear side of p-n junction and principal component analysis of PV signals allows identifying mentioned liquid substances and their mixtures.

## Background

Nowadays, the research and development of sensory systems like lab-on-a-chip [1, 2] for monitoring of toxic compounds in the atmosphere, water, food, and the identification of chemical composition of the outer space at all are becoming important task. The use of semiconductor chemical sensors is the promising way to realize the lab-on-a-chip conception. However, the problem of the low selectivity makes the semiconductor sensor application very complicated. One of the approaches in solving this problem is by using of large arrays of low selective semiconductor sensors. It appeared during the researches of mammalian senses. Biological explorations show that high sensitive and selective mammalian organs of taste and smell consist of many low selective receptors of different types [3]. Such great selectivity is explained by the advanced signal treatment that is gained from the receptors’ array by the peripheral and central nervous system.

That is why, an idea of creation of an “electronic nose” and “electronic tongue” (low selective sensor arrays that could be artificial analogues for organs of taste and smell) is widely discussed [4–7]. Such electronic devices consist of two parts—chemical sensor and mathematical treatment blocks. The selectivity of these devices is mainly determined by mathematical methods and algorithms. The other approach for solving the non-selectivity problem is the considering of the specific adsorbate–adsorbent interaction, application of catalysts, using ion selective membranes and electrodes, etc. [3]. However, this approach decreases sensors’ universality and increases the influence of surroundings on the measurement accuracy. There are no such problems for the electronic nose and tongue. They could be multipurpose, i.e., could identify many different substances and even analyze the content of a multi-component medium.

In this work, we present an experimental verification of new conception of an electronic tongue on the base of an array of low selective sensors that produce light-induced photovoltaic signals (PV). The approach uses the idea that the induced PV signal of p-n junction should noticeably depend on the influence of the ambient molecules [8, 9] on the surface of the structure. Before, the influence of p-n junction depth, doping level, diffusion length on photocurrent of planar silicon junction for different value of surface recombination was studied by numerical simulation [9]. It allows optimizing the junction parameters from point of view of its efficient application as a chemical sensor with PV transducer.

## Methods

The electronic properties of interface are strongly affected by molecule absorption if nanostructured layer is created on the silicon substrate since nanocrystalline silicon effectively absorbs several types of molecules [10]. The response of each sensor (random spot on surface) to the tested substance (including the adsorbate–adsorbent interaction at various experimental conditions) differs from such response of other sensors of the array due to the natural dispersion of surface recombination rates. However, the selectivity of each of these sensors is too low to determine an unknown substance. Though the responses of each of low selective sensor depend insignificantly on the type of adsorbing substance, a set of numerous responses creates a primary fingerprint of the substance. That is why, an array of such elementary sensors and their responses has to be formed and information from it should be evaluated mathematically using pattern recognition technique. This approach gives a hope to create highly selective sensor devices.

A sensor array was formed on the base of shallow (0.5 μm of depth) p–n junctions on crystalline p–Si (4.5 Ω/cm) substrates. A porous silicon layers of ~100-nm thick and of 60 % porosity were grown electrochemically on p-base side to ensure access of foreign molecules and testing light. A scheme of the sensor structure was displayed in [9]. The photocurrent of such solar cell contacted with different liquids was obtained by the conventional light beam-induced current (LBIC) technique [11]. The structures were illuminated from the rear side by modulated red light (*λ* = 0.63 μm) from He–Ne laser (at modulation frequency of 1276 Hz) to obtain 2D map of light-induced signal. The laser beam scanning was provided due to acousto-optic deflector. The light spot was moved systematically on the sample surface, addressing discrete spots forming 256 × 256 matrix. Therefore, the data were received from different spots due to the light addressing. The photocurrent was registered by selective nanovoltmeter Unipan-237 model. The obtained signal was processed and analyzed by PC.

During scanning, the individual response of the illuminated spot appears independently of other area. Therefore, the conception does not need (but does not discard) an array of separate elements. The user of the device determines the quantity, the layout of the elements to be read. With the same device, it can be variable. The possibility to use a substrate without physical separation by individual elements, i.e., “dynamical array” with variable parameters may be considered as an advantage.

## Results and Discussion

**Fig. 1:**
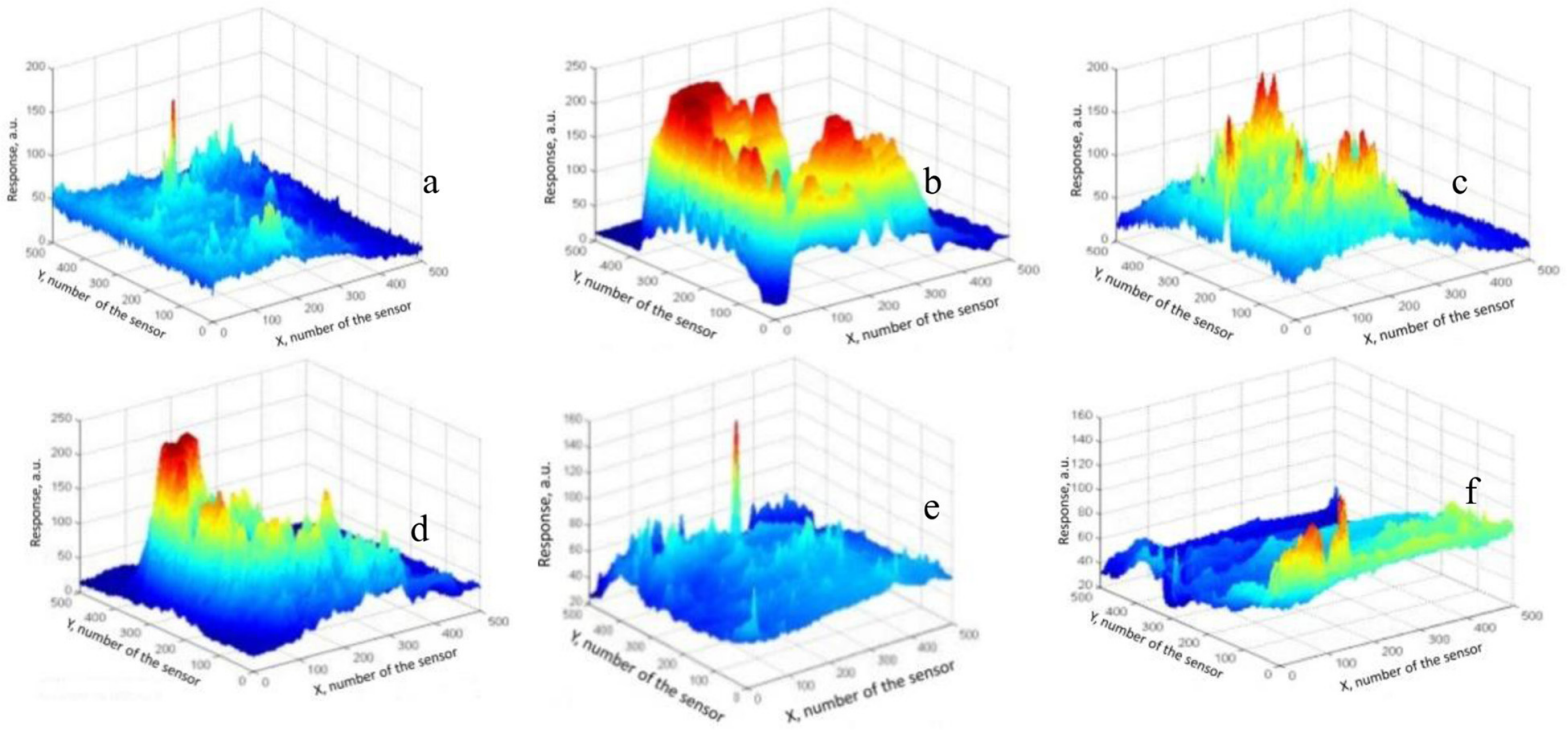
Distribution of PV signal *S(x,y)* on the surface of PV transducer (3D maps) under the influence of: water (**a**) iso-propanol (**b**), ethanol (**c**), and their mixtures: ethanol + iso-propanol (**d**), water + ethanol (**e**), water + iso-propanol (**f**) (*x,y*—two-dimensional coordinates that define the position of sensors in the matrix)

We have carried out experiments to check the proposed possibility how the mentioned optoelectronic sensor structure can realize a function of multisensor matrix. Figure 1 represents such maps under influence of different liquids. The observed PV response is inhomogeneous and depends on the type of influence. Therefore, it is possible to consider the local responses of the sensor structure as responses from different sensors.

In conventional solar cells, p-n junction is situated close to the illuminated surface. So, the photogenerated electron–hole pairs are separated immediately by strong electric field of the p-n junction and produce a photocurrent. In other words, a chance to be caught by p-n junction is much higher than to be captured by surface recombination center. In the case of our device, p-n junction is situated on the opposite to the illuminated surface. Only small part of the photogenerated carries get the p-n junction due to diffusion process (and produces photocurrent), but most of them recombine within the illuminated surface and volume of the semiconductor substrate. Therefore, the photocurrent strongly depends on the surface recombination velocity which could depend on the adsorption, surface reactions, etc. So, the determinative feature of this sensor device is believed that surface recombination channel becomes competitive with separation function of p-n junction due to relocation of the p-n junction from the face surface of the device.

To comply with the approach of the light addressed sensor matrix, the induced PV signal of mentioned elementary sensors should noticeably depend on the influence of ambient molecules. The electronic properties of the silicon surface are known to be strongly affected by the liquid (gas) absorption [8]. It realizes the principle that the photocurrent collected by p-n junction depends on recombination velocity if the incident light is absorbed close to the reactive interface. It was shown by simulation with the help of PC-1D program that in such device the photocurrent induced by 0.63 μm red light falls by 2–3 orders times when the surface recombination velocity increases in its practical range from 10^3^ to 10^5^ cm/s [9]. Thus, the photocurrent observed in this configuration is an express measure of the interface status and considered as the sensing parameter. As seen from Fig. 1, the observed sets of PV responses depend on the adsorbate’s type. Thus, they can serve as fingerprints (patterns) for substances.

We suggest two physical mechanisms of such influence. Firstly, the exposition of the sensor surface to the foreign molecules may change the electrical charge located in the interface [12]. Thus, the change of band bending drastically influences on the recombination conditions. Other mechanism includes the appearance of new recombination centers associated with the adsorbed molecules [10]. Concerning the PV signal, the recombination reaches the maximum when *p*_*s*_/*n*_*s*_ = *σ*_*e*_/*σ*_*h*_ [13], where *σ*_*e*_ and *σ*_*h*_ are electrons and holes capture cross sections; *p*_*s*_ and *n*_*s*_ are holes and electrons concentration at the surface.

The presence of the sensitive layer of porous silicon significantly expands the potential for sensor research not only in liquids but also in vapors of various liquids too. As is known, the adsorbate vapor can condense, which is described by the well-known Kelvin formula:

$$ \mathrm{In}\frac{P}{P_0}=\frac{2\sigma {V}_m}{aRT} $$

where *P* is the actual vapor pressure, *P*_0_ is the saturated vapor pressure, *σ* is the surface tension, *V*_*m*_ is the molar volume of the liquid, *R* is the universal gas constant, *a* is the radius of the pore, and *T* is the temperature.

Calculation shows that at the adsorption, for example, water vapor into porous silicon with 3 nm pore radius, the condensation occurs at relative humidity RH, (RH = 100*P*/*P*_0_) = 75 %since *σ* = 7.3 × 10^−2^ N/m, *V* = 1.8 × 10^−5^ m^3^/mol at room temperature. Therefore, the presence of a porous material in the sensor allows to investigate complex mixtures of vapor effectively that is problematic for a flat surface structure.

Besides the mentioned requirement of the noticeable dependence of each sensor response on the kind of acting adsorbates, these responses from different sensors of the matrix should be different (even for the same adsorbate). Moreover, to create the initial set of sensor responses suitable for the further pattern recognition, the sensors have to form their responses under various experimental conditions (such as temperature, illumination, etc.). This approach may enhance the useful information from the matrix of sensors. In turn, each response of the certain sensor corresponding to the conditions of its measurement should depend on the acting substance.

As shown in Fig. 1, there were 256 × 256 = 65536 sensors in our experiment, but there was no need to use all of them. At first, signals from many sensors were nearly identical for one measurement and identical or linearly dependent for repeated measurements. Such signals do not increase the self-descriptiveness of the sensor matrix. Secondly, the mathematical treatment for so many signals requires a lot of computational resources. That is why, sensors with different signals should be chosen. The minimal number of “working” sensors must be enough for forming the statistical sampling and not be less than the number of substances, which sensors had to identify.

**Fig. 2:**
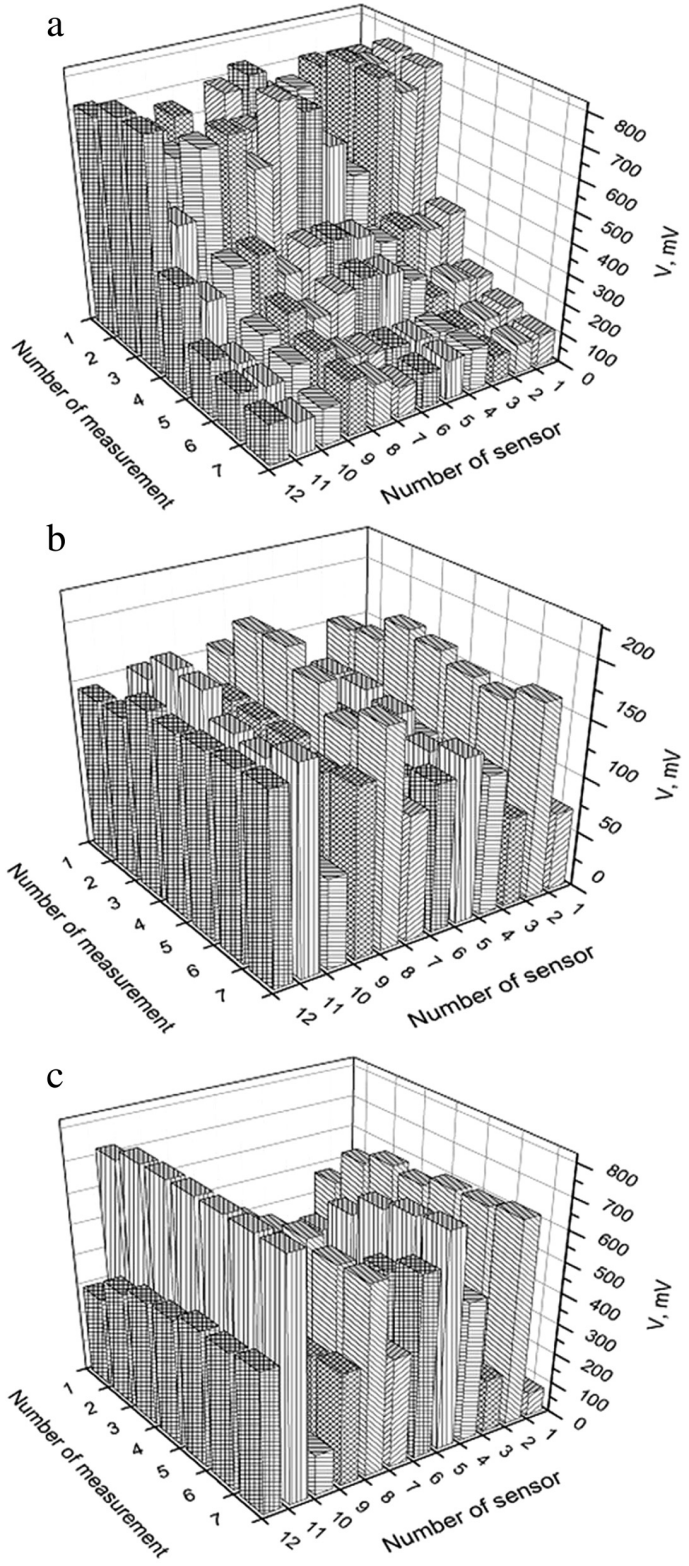
The responses of the sensors for three different adsorbates (water (**a**), ethanol (**b**), iso-propanol (**c**)) measured at time points with 2-min interval

From such 3D maps, several points were chosen and the laser testing illumination was directed for these points only. In such way, the time depended behavior of the PV respond was measured for these virtual sensors under different influences. As can be seen, the sensor signals are varied significantly in different liquids (Fig. 2). Nearly the same results are obtained at the repeated measurements (3D map for every substance can change a little, but the main features of it (for example, position, amount, and maximums’ amplitude are similar), i.e., the sensors show nice stability during measurement cycle. It needs 20 s to measure one virtual sensor kinetics. In our experiment, 12 virtual sensors (points within the sensor area) were tested, and these measurements were repeated seven times with 2-min interval, and the same procedure was for six different types of influences (water, ethanol, iso-propanol, water/ethanol, water/iso-propanol, ethanol/isopropanol). Thus, 12 × 7 × 6 = 504 files of PV signal kinetics were measured and saved. Both signal amplitude and kinetics vary from sensor to sensor and depend on the type on influence.

Figures 1 and 2 show nice selectivity of the proposed sensor structure: there is a strong correlation between PV signals for each adsorbate. Such forms of the initial data can already serve to distinguish different adsorbates. However, this method of substances’ recognition requires visual expert estimation of these diagrams.

Different mathematical methods could be applied to identify substances according to the response of the proposed sensor structure. The most advanced and universal of them are the methods of pattern recognition technique. The term “pattern recognition” can be defined as the transformation of an input data array (for example, responses from the sensor matrix) to an output data array (for example, the type of adsorbates, their concentration). Principal component analysis (PCA) [14] is one of the most promising, all-purpose, and simple methods of the unsupervised pattern recognition technique for the sensor application.

PCA could be considered as a “black box” that extracts some hidden features in the input data array and calculate new output data array that describes substances. The main geometrical aim of PCA is finding of the most optimal (in terms of explained dispersion) projection of the input data array, which can be represented in multidimensional space as the set of points, onto a space of reduced dimensions (space of principal components). Usually, it has two or three dimensions, so it can be represented geometrically. Distances between points in the space of principal components correspond to their likeness, so compare and classify them. These points belong to the sensor signals. That is why, this approach gives the possibility to compare signals and thus identify compounds.

**Fig. 3:**
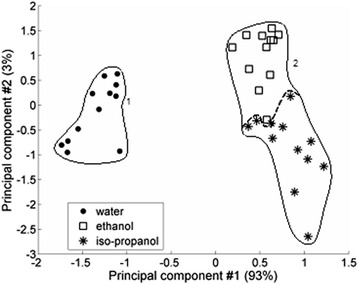
The comparison of the PV transducer response under the influence of water, ethanol, and iso-propanol

The input data array (matrix) is formed from signals *V(t)* that were used to build Fig. 3. Classically, the input data matrix *Z = (z*_*ij*_*)* has *m* rows and *n* columns, where *m z*_*i*_ (moments of time for this experiment), *n*—the number of objects (sensors) that are investigated. Usually, the input data matrix is standardized before its using. (The standardization of the matrix *Z* sets for its every variable *z*_*i*_ the mean to 0 and the variance to 1.) PCA is one of the factor analysis methods and, at the same time, one of the most widespread methods of analyzing a multidimensional data (chemometrics) [15–17].

Our main aim is obtaining a possibility of the “predictive” comparison of the signals *V(t)* for identifying substances. One of the most simple and simultaneously universal ways of solving is the representation of every signal *V(t)* (therefore, every sensor that gives it), which is stored in the input data matrix, as the point of the space of measurements (space of variables). Every sensor could be represented as a point of the seven-dimensional space (since the repeated measurements are occurred for seven times). Points’ clustering in this space will compare and classify them, so it may solve the problem of substances’ identification. The amount of clusters will determine the number of different substances. Unfortunately, the pictorial rendition of such space is impossible due to its high dimensionality. PCA gives the possibility to decrease the dimensionality of the variables’ space and to change it by the space of principal components. Let us consider this question in detail (demonstrably, not mathematically precisely).

According to the base principles of chemometrics, every measurement includes both important (useful) and unimportant information (the noise) [17]. Important information is all those signals features that allow identifying substance and characterize it. For example, there is a monotonic decay on all dependences *V(t)* for water. The noise is caused by incomplete reproducibility of measurements, insufficient purity of the investigated substances, and, maybe, by the unknown processes on the surface.

It is logical to expect that in our experiment, the signals *V(t)* have to be identical for each substance because our sensor structure consists of the same sensors. However, Fig. 1 shows the presence of some differences in signals’ form. The main factors that cause these differences are the heterogeneity of the surface and the noise. Nevertheless, such differences cannot hide some significant regularities in signals *V(t)* for each substance that assist to identify it.

Theoretically, only one sensor could be used to compare adsorbates, but such comparison will be too inaccurate because in this case, it is impossible to distinguish the noise from the useful information in signals. Therefore, the matrix of sensors is used. They form the representative statistical sampling that gives the possibility to discriminate correlations between signals (or more exactly, at first, correlation between variables) for each substance, i.e., to compare adsorbates.

So, we can represent signals-sensors in the multidimensional space of variables, but pictorial rendition of this space is possible only when its dimension is *d* ≤ 3. For our experiment, *d* = 7. The only possible way of solving the geometrical interpretation problem is the decreasing of dimensionality without losing much useful information from the sensor structure (information about points’ clustering for our work). But before the realization of the dimension decreasing procedure, we should introduce the mathematical criterion of the self-descriptiveness. We should explain what information has to be saved, what information is the most important. The self-descriptiveness in PCA closely corresponds to the dispersion of variables from the input data matrix.

**Fig. 4:**
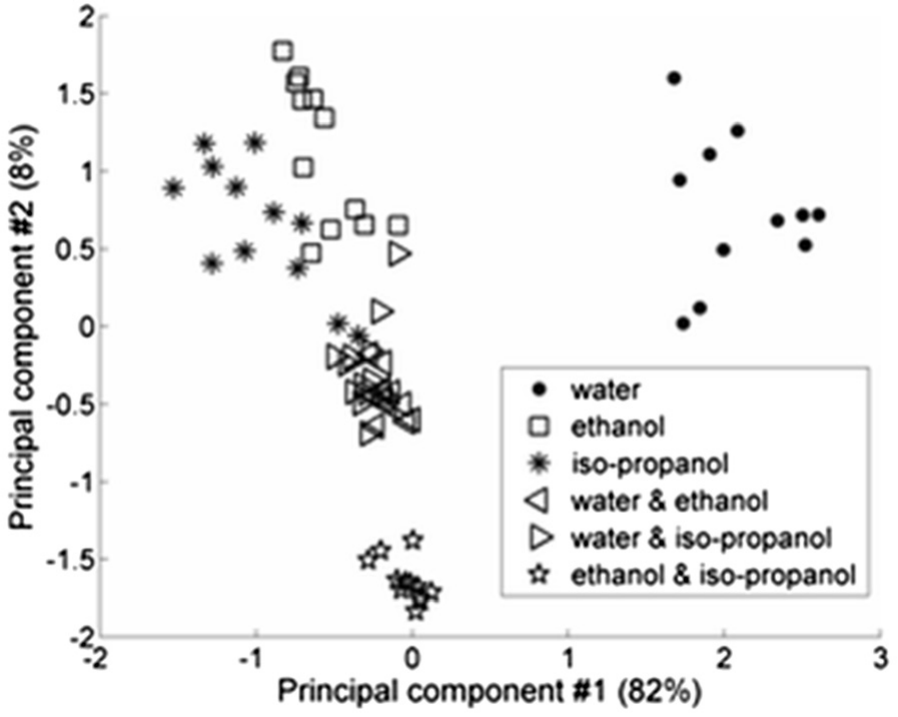
The comparison of the PV transducer response under the influence of water, ethanol, iso-propanol, and their mixtures

After obtaining signals *V(t)* for 12 sensors under the influence of different liquids, these signals should be written to the matrix of input data, and it should be processed with PCA to find the matrix *F*, which the graphs of factor scores had to be plotted. The comparison of sensors’ signals under the influence of water, ethanol, and iso-propanol is shown on the graphs of factor scores (Fig. 3). Herein, we have to notice that usually, there are no dimensions on the axes of factor scores’ graphs. To understand that, we have to recollect that principal components (as variables) are the linear combination of variables *z*_*i*_ (and vice versa) that have different dimensions in general. Every “point” in Fig. 3 represents the sensor. Different form of labeling for groups from 12 sensors was used to show that these sensors were applied for measurements under the influence of different substances. Groups of sensors that were calculated using the hierarchical cluster analysis [18] are circumscribed by geometrical figures. Figure 3 shows that the mentioned sensor structure is capable to distinguish water and alcohol and in less extend ethanol and iso-propanol that confirms their common chemical nature. The first two principal components describe more than 90 % of the total variance of the matrix *Z*. That is why, the first two principal components are enough to explain all useful information from the sensor structure and to distinguish investigated substances [19, 20]. The final comparison of sensors’ signals under the influence of all our tested substances and their mixtures is shown on Fig. 4.

## Conclusions

An experimental verification of new conception of an electronic tongue on the base of an array of low selective sensors that produce light-induced photovoltaic signals is presented. The determinative feature of the studied sensor device is believed that surface recombination channel becomes competitive with separation function of p-n junction due to relocation of the p-n junction from the face surface of the device. A dynamical array of such photovoltaic sensors with optical addressing and dispersion of their parameters due to natural heterogeneity of their surface is shown to build the selective electronic tongue. The presence of the sensitive layer of porous silicon significantly expands the potential for sensor design, not only in liquids but also in vapors of various liquids too; it improves the sensitivity significantly and possibly contributes the necessary cross-selectivity. Principal component analysis compatible with the sensor structure and data acquisition process was demonstrated to find some hidden correlations of such low selective sensor responses to distinguish substances and their mixtures.

## Abbreviations

LBIC: Light beam-induced current
PCA: Principal component analysis
PV: Photovoltaic
